# Neural network modelling of proton RBE values at predominant survival fractions of in vitro data

**DOI:** 10.1038/s41598-026-54123-w

**Published:** 2026-05-22

**Authors:** Erlend Lyngholm, Armin Lühr, Liheng Tian, Camilla Hanquist Stokkevåg, Helge Henjum, Andreas Havsgård Handeland, Johannes Tjelta, Kristian Smeland Ytre-Hauge

**Affiliations:** 1 Department of Physics and Technology, University of Bergen, Bergen, Norway; 2 Department of Physics, TU Dortmund University, Dortmund, Germany; 3 Cancer Clinic, Haukeland University Hospital, Bergen, Norway

**Keywords:** Radiotherapy, Biological physics, Translational research

## Abstract

While a constant relative biological effectiveness (RBE) of 1.1 is applied in clinical proton therapy, longstanding clinical concern and *in vitro* evidence demonstrate a variable proton RBE. Several RBE models for protons have been developed based on general assumptions of RBE dependencies together with fitting to *in vitro* data, for which some modelling authors have included greater diversity of cell types than others. In this study we use neural networks (NNs) to model the proton RBE without any predefined dependencies, investigate the effect of using different biological inputs and combinations of input parameters in the modelling, and compare the results to previous approaches. A comprehensive *in vitro* RBE database of 431 data points was used, including parameter values for the proton linear energy transfer (LET), the (α/β)_x_ value of the linear quadratic model parameters of the reference photon radiation, and survival fraction (SF)-specific photon doses (D_x_(SF)). NNs were used to model RBEs at specific SFs (RBE_SF_) as a function of (1) LET, (2) LET and (α/β)_x_ and (3) LET, (α/β)_x_ and D_x_(SF). Models with inputs LET, (α/β)_x_ and D_x_(SF) showed overall best performance, particularly inputs with D_x_(0.1) and D_x_(0.2), indicating that an appropriate standardized input parameter for D_x_(SF) could be established by choosing an SF between 0.1 and 0.2. The presented approach to assumption-free NN-based modelling of proton RBE showed similar RBE dependencies as previously published models with similar predictive power. Further, it was demonstrated that adding the photon dose sensitivity as an input parameter to modelling could increase the quality of RBE models.

**Supplementary Information:**

The online version contains supplementary material available at 10.1038/s41598-026-54123-w.

## Introduction

A constant relative biological effectiveness (RBE) of 1.1 remains the foundation of clinical proton therapy practice today. The RBE is defined as the ratio of a reference photon dose versus the proton dose to reach the same level of biological effect for a specific endpoint. An error in RBE in clinical applications therefore leads to a biological radiation effect in patients that deviates from the effect intended in treatment planning. The simplistic proton RBE value of 1.1 was adopted based on limited early *in vivo* experimental data, primarily from short-term biological assays in the 1970s, without detailed mechanistic or spatial analysis^1^. It was introduced as a conservative, pragmatic standard to support safe clinical implementation rather than precise biological accuracy. However, the use of this generic RBE factor has been questioned for several decades^2,3^, and there is now solid *in vitro* evidence of a variable proton RBE, which has also been observed *in vivo* and clinically in patients^4–7^. Most published proton RBE models rely on regression fits to *in vitro* RBE data of fitting functions for RBE_max_ and RBE_min_, which correspond to RBE values at extreme dose limits^8–16^. Numerical values for RBE_max_ and RBE_min_ are derived from the linear quadratic (LQ) model parameters of the proton radiation (α, β) and the reference photons (α_x_, β_x_) calculated as α/α_x_ and $\:\surd\:$(β/β_x_), respectively^17–19^. The fitting functions are generally also based on assumptions regarding RBE dependencies on the linear energy transfer (LET), and the photon LQ parameters. The values of α_x_ and β_x_ quantifies the intrinsic cellular radiosensitivity, reflecting the genetic susceptibility of cells to the damaging effects of radiation. The reference photon LQ parameters have been established as important predictive parameters for RBE modelling, and the quotient of α_x_ and β_x_, (α/β)_x_, is commonly used as a measure for the fractionation sensitivity of different tissues and organs. Many extant models use the (α/β)_x_ value as the basis of the mathematical construction, linked to the gradient of RBE with LET, aiming to account for variations in RBE between different tissues and endpoints^20^. Besides using RBE_max_ and RBE_min_, alternative approaches have been proposed to parametrize proton RBE dependencies using different candidate functions^21^ or fitting mechanistic models to the data^22^.

Unlike the above-mentioned methods, machine learning (ML) enables creation of models without relying on specific assumptions or candidate functions. Both Tian and Lühr^23 ^and Cordoni et al.^24^ demonstrated that ML could be used to create generalized RBE models for ions based on *in vitro* data from a range of ion species. However, heavier ions extend to LET ranges far beyond those of protons, highlighting the need for dedicated RBE modelling for protons. Furthermore, as cell irradiation experiments are limited to specific dose ranges, corresponding to a confined range of survival fractions, using RBE_max_ and RBE_min_ assumes that the LQ model fits remain valid beyond these ranges, thus representing an extrapolation of the data.

To explore the proton RBE without predefined model assumptions or candidate functions while also addressing the limitation of extrapolation related to RBE_max_ and RBE_min_ we performed neural network (NN) modelling of a proton *in vitro* database, estimating RBE at specific survival fractions covering the predominant ranges within the available raw data. In addition to LET and (α/β)_x_, photon doses at specific SFs were used as input in the models to determine whether including this parameter provides additional insights that could contribute to a better understanding and modelling of the proton RBE.

## Materials and methods

This study utilized a previously published comprehensive proton *in vitro* RBE database^16^ containing data obtained with both monoenergetic and spread-out Bragg peak beams. Each data point includes proton- and reference photon LQ parameters (α, β and α_x_, β_x_, respectively) and dose-averaged LET (LET_d_) for the proton irradiation normalized to the LET_d_ of Cobalt-60 to account for the variation in reference radiation quality across different experiments (denoted LET*, see Supplementary material for more details)^16^. More detailed information about each data point can be found in Lyngholm et al.^16^, where the database was originally collected and provided. An LQ fit to experimental raw data can result in negative LQ parameters. However, a negative alpha term does not align with its mechanistic meaning^25^, which is why such data were omitted. A negative beta term can occur, e.g., if subpopulations with different radiosensitivities coexist within the cell culture^25^. Therefore, such data were also omitted to only consider data for cell cultures with a uniform radiation response. Since α_x_ or β_x_ equal to zero result in (α/β)_x_ values of zero or infinite, respectively, data with such photon LQ parameters were omitted as well. For these reasons, only data with positive photon LQ parameters and α and β values ≥ 0 were considered. Furthermore, the database was restricted to only include data points with LET* ≤ 20 keV/µm and (α/β)_x_ ≤ 20 Gy.

**Fig. 1:**
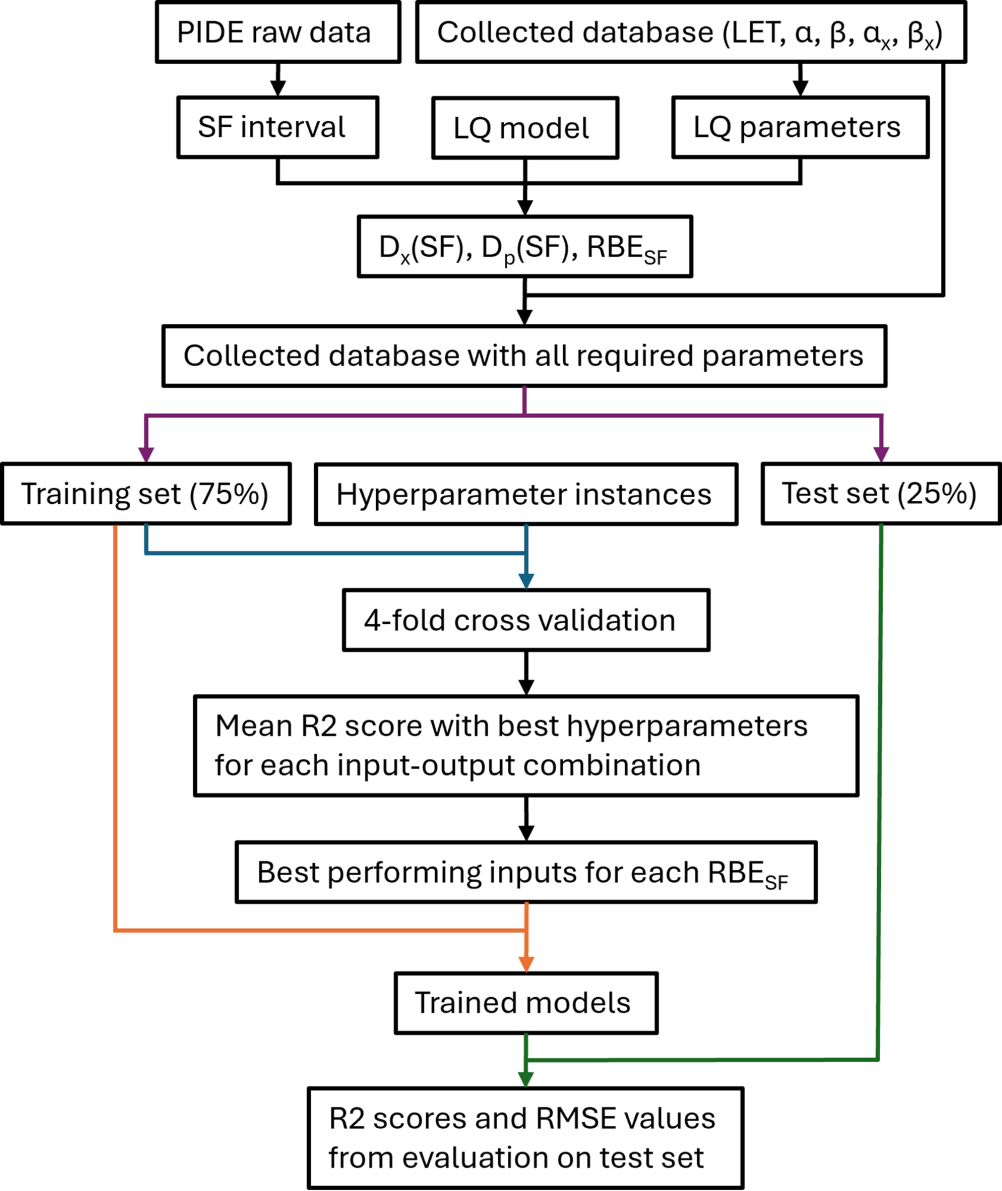
Flowchart showing the workflow of the methods utilized for the analysis performed in the current study.

The Particle Irradiation Data Ensemble (PIDE) database version 3.2^25,26^ provides raw data of proton- and photon survival experiments, i.e. the measured points of doses and corresponding survival levels from which LQ parameters can be obtained through LQ model fitting. The experimental raw data were used to determine a suitable survival range for inclusion in the current analysis, i.e. a range covered by most experiments to minimize extrapolation and simultaneously being wide enough to model RBE values at distinctly different SFs. The assumption was thus made that the SF interval determined from this raw data could be generalized to apply for our database, also containing data beyond PIDE. An overview of the methodology for modelling of RBE values at specific survival levels is shown in Fig. 1.

For each data point in the database, reported values for α_x_, β_x_, α and β were inserted into the LQ model to calculate the photon- and proton doses at survival fractions equal to 0.1, 0.2, 0.3, 0.4, 0.5, 0.6 and 0.7, denoted as D_x_(0.1), …, D_x_(0.7) and D_p_(0.1), …, D_p_(0.7), respectively. Corresponding RBE_SF_ values for each survival fraction (RBE_0.1_, …, RBE_0.7_) were computed as the ratio D_x_/D_p_, according to the definition of RBE. These RBE_SF_ values were considered as the experimentally derived ground truth in this work.

The data were randomly separated into training- and test sets containing 75% and 25% of the data, respectively. A NN regressor (MLPRegressor, scikit-learn^27^ was used to model RBE_SF_ values as a function of (i) LET, (ii) LET and (α/β)_x_, (iii) LET and D_x_(SF) and (iv) LET, (α/β)_x_ and D_x_(SF). The NN model approach was chosen to avoid any assumptions for RBE dependencies on the input parameters, which would have been a necessity for regression fitting to predefined fitting functions. For each RBE_SF_, the selection of model settings was made by using cross validation on the training set to determine the best performing hyperparameters and model inputs. The parameters for the selected model settings were then fitted to the entire training data. The prediction power of the resulting trained RBE_SF_ models was evaluated using the test set (Fig. 1).

The training set was modelled for each of the inputs/output combinations according to a 4-fold cross validation scheme using different hyperparameter-instances. The considered hyperparameters in the MLPRegressor function were the number of hidden layers and number of neurons in each of them (*hidden_layer_sizes)*, the activation function for the hidden layers (*activation*), the algorithm used for weight optimization during training (*solver*), the strength of the L2 regularization term which helps avoid overfitting by preventing any one weight from dominating the model (*alpha*), and the integer value determining the random number generation for weights and bias initialization as well as batch sampling and assuring reproducible results (*random_state*). The input values investigated for each hyperparameter are given in Supplementary Table A2. The out-of-sample adjusted coefficient of determination (see Supplementary Equation (A6)), which is referred to as “R2 score” for simplicity in the following, was used as performance measure in the cross validation. This variant of the coefficient of determination is modified to (1) take into account the number of input variables in the model, by penalizing extra variables which do not significantly improve the model and (2) measure the performance of a model exclusively on data it has not seen during training. For each modelled combination of inputs and output, the hyperparameter instance that resulted in the highest mean R2 score across the four folds was used as the final hyperparameters for this model. Among the 16 alternatives for inputs used in the modelling (only LET* (1), LET* and (α/β)_x_ (1), LET* and D_x_(SF) (7) and LET*, (α/β)_x_ and D_x_(SF) (7)), the best performing ones for each RBE_SF_ were determined based on highest cross validation mean R2 scores. For each RBE_SF_, the inputs that yielded best performance in the cross validation were used to fit the model with the corresponding hyperparameters to the training data, resulting in one trained NN model for each of the seven SF-levels. The prediction power of each model was evaluated on the test set, calculating the R2 score between the predicted and experimentally derived RBE_SF_ values.

The NN models were also compared to the previously published Wedenberg model (WED)^14^, McNamara model (MCN)^11^, and Lyngholm model (LYN)^16^, which were implemented as presented in the original publications. The WED and MCN models were used for comparison as they are both frequently used in recent publications investigating potential RBE effects^28–34^, while the LYN model is currently the LQ-based phenomenological model that is based on the largest database^16^. All three models were also developed using data from multiple cell lines, as opposed to several other models which included less diversity of cell types in the model databases^20^. RBE_SF_ predictions of these models on the test data were obtained using the corresponding D_p_(SF) values as dose input in addition to LET and (α/β)_x_. Root mean squared errors (RMSEs) between predicted and experimentally derived values were calculated for these models as well as the seven NN models developed in the current study. For visual comparison, 3D plots were produced showing RBE_SF_ as a function of LET and (α/β)_x_ according to each model. The dose inputs (D_x_(SF) in the NN models and D_p_(SF) in the previously published models) were kept at constant values equal to the average of all input values for the corresponding parameter in the database.

## Results

**Fig. 2:**
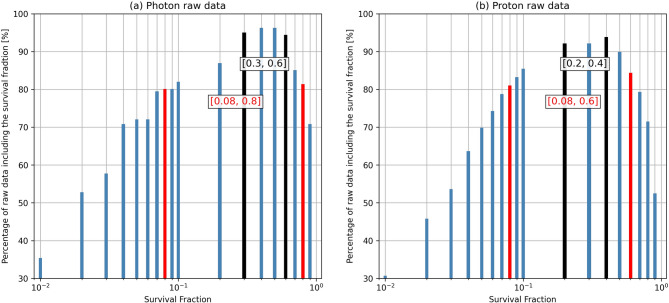
Bar plots showing the percentage of data covering different survival fractions for the photon- (**a**) and proton (**b**) raw data provided in the PIDE database. The bar at each SF gives the percentage of data points for which this SF is included in the SF range covered by the corresponding raw data. The red and black bars indicate the upper- and lower limits of the SF intervals covered by 80% and 90% of the data, respectively, which are given in text boxes with writing in corresponding colors.

Among the 180 proton data points included in PIDE, photon raw data had not been reported for 19 data points^35–38^, while proton raw data was available for all except one^36^. As can be seen from the bar plots in Fig. 2 showing the SF coverage of the PIDE raw data (used to derive α_x_, β_x_, α and β values), 80% of the photon experiments cover the SF interval [0.08, 0.8], while the corresponding interval for proton irradiations is [0.08, 0.6]. For 90% data coverage, the SF intervals are much narrower: [0.3, 0.6] for photons and [0.2, 0.4] for protons. It was decided to use SFs in the interval [0.1, 0.7], as 79% of the proton raw data cover survivals up to 0.7, ensuring that the modelled SF range includes minimal extrapolation beyond the measured raw data while not being too narrow.

The proton *in vitro* database contained 434 data points in total. However, an assessment of potential outliers led to the exclusion of three data points: One data point from Ristic-Fira et al. 2008^39^ showing significantly higher dose values for the considered SFs compared to the rest of the data. Two data points given by Bronk et al. 2020^40^ were considered unsuitable for the current analysis as RBE values at SFs of 0.1 and 0.5 were missing in the original publication due to lack of data coverage.

**Fig. 3:**
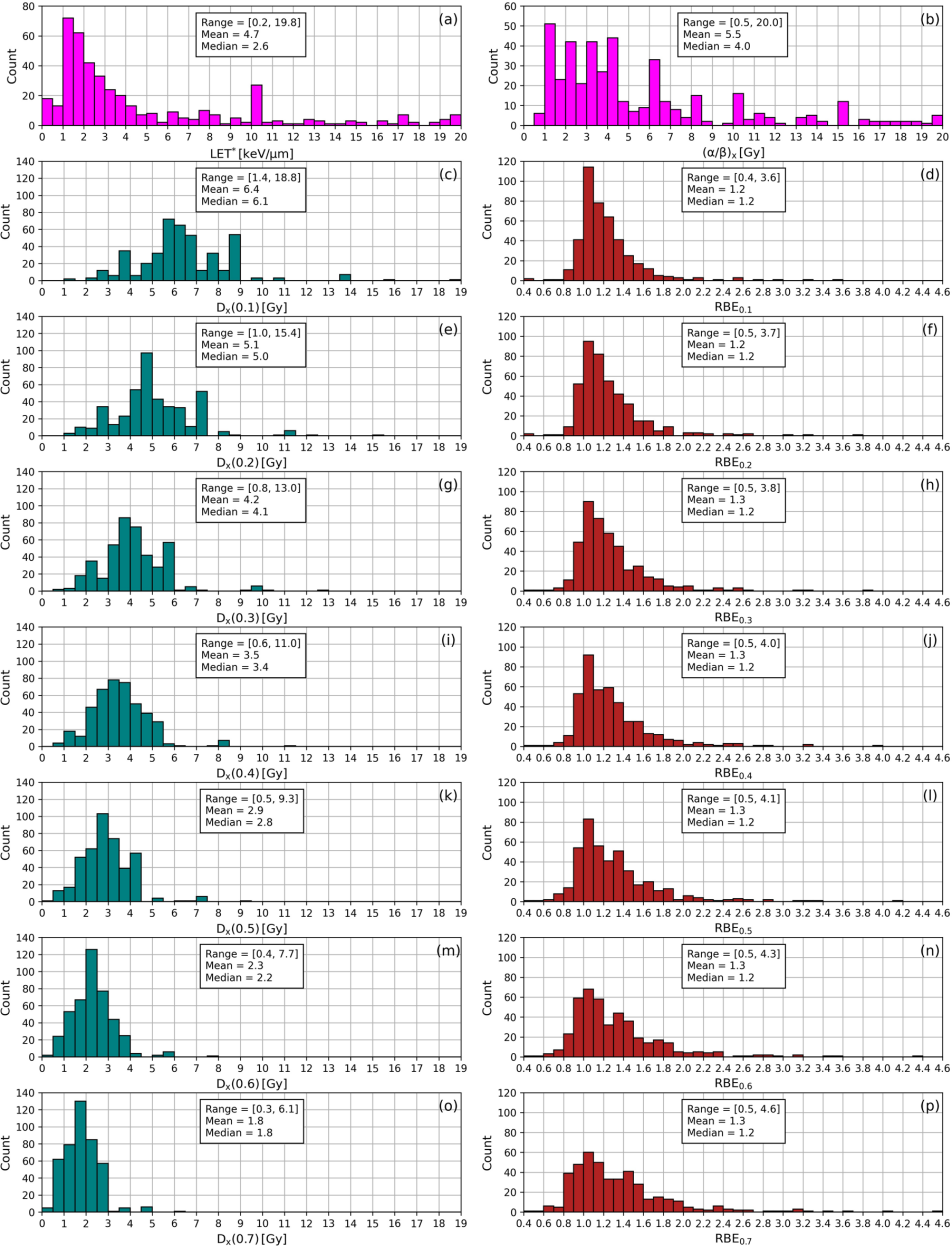
Histograms showing the distributions of LET and (α/β)_x_ values for the 431 proton data points are given in panels **a** and **b**, respectively. Corresponding distributions of D_x_(SF) and RBE_SF_ values are displayed in panels **c**-**p** for SFs of 0.1 (**c-d**), 0.2 (**e-f**), 0.3 (**g-h**), 0.4 (**i-j**), 0.5 (**k-l**), 0.6 (**m-n**), and 0.7 (**o-p**). In each panel, the range of parameter values in the database, as well as their mean and median values, are provided in text boxes.

**Fig. 4:**
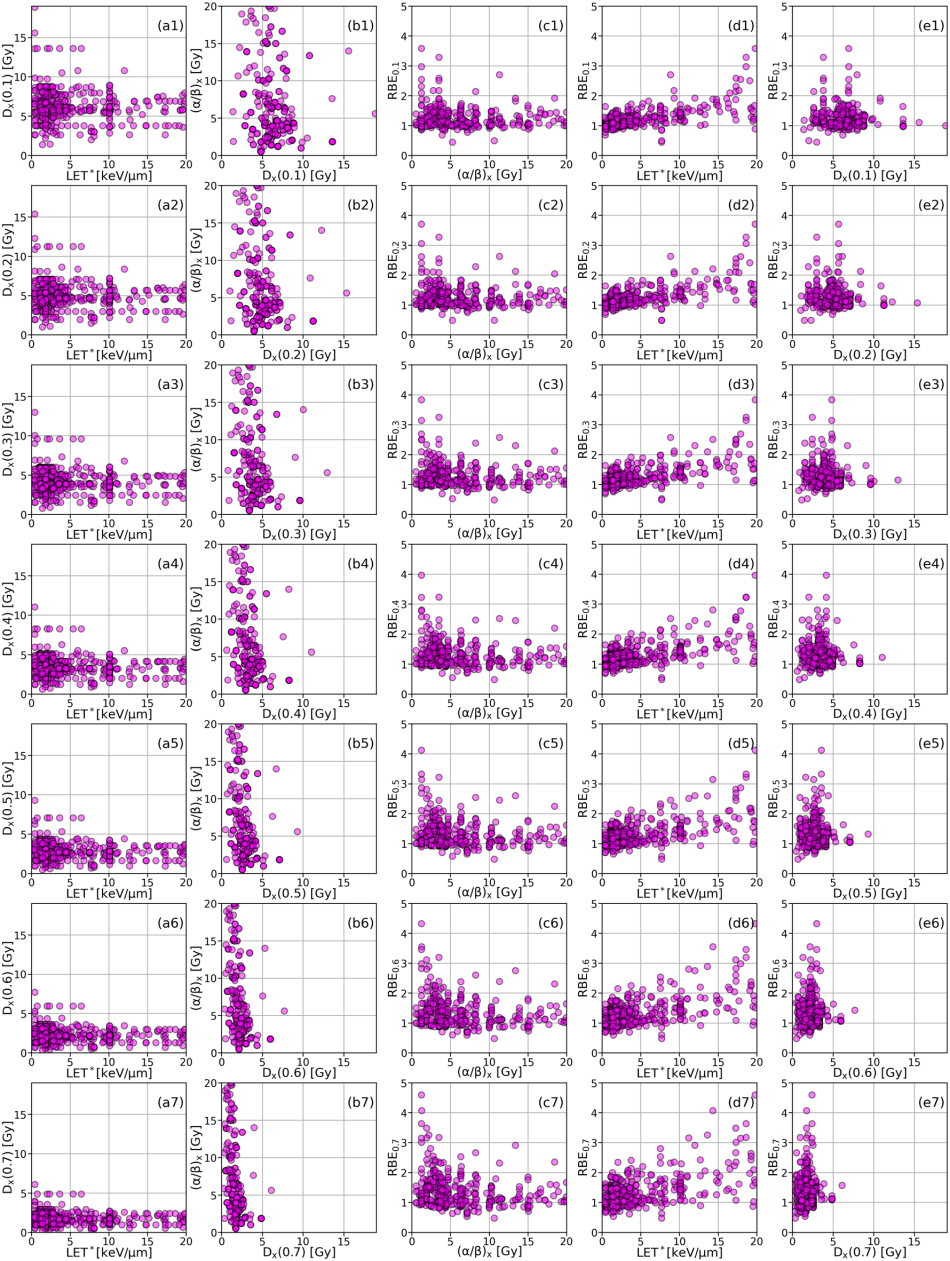
Scatterplots of LET vs. D_x_(SF) (**a1-a7**), D_x_(SF) vs. (α/β)_x_ (**b1-b7**), (α/β)_x_ vs. RBE_SF_ (**c1-c5**), LET vs. RBE_SF_ (**d1-d7**) and D_x_(SF) vs. RBE_SF_ (**e1-e7**) are shown for all considered SFs between 0.1 (first row, **a1-e1**) and 0.7 (last row, **a7-e7**). Each plot includes all 431 data points in the database.

The database that was used in the following thus contained 431 proton *in vitro* data points, including 106 from PIDE. The data covers an LET range between 0.24 and 19.8 keV/µm and (α/β)_x_ values from 0.51 Gy to 20.0 Gy (Fig. 3). The ranges of D_x_(SF) and RBE_SF_ values for each considered SF are given in Fig. 3, and we see that for increasing SF the prior becomes narrower while the latter becomes wider, which is also seen in the right column in Fig. 4, panels e1-7. The distributions of LET and (α/β)_x_ were clearly imbalanced (Fig. 3 panels a and b), with a larger portion of the data having values in the lower part of the ranges covered by the data. Plotting LET vs. (α/β)_x_ (Supplementary Figure A1) also shows that most of the data had both low LET and (α/β)_x_, and the regions with low LET and higher (α/β)_x_ or the other way around were also relatively well covered, while the data was much more sparse for both higher LET and (α/β)_x_, in the region limited by roughly (α/β)_x_ > 5 Gy and LET > 5 keV/µm.

The plots in Fig. 4 show the regions of the data space that are more densely populated, i.e., where we can expect models trained on this dataset to be more stable and perform consistently well on new data. From the rightmost column in this figure, panels e1-7, the D_x_(SF) values seems to be mostly clustered together with a sharp fall in the amount of data outside a certain range which becomes narrower and moves towards lower doses for higher SFs. For example, for SF = 0.1 this range seems to be roughly 2.5–9 Gy from Fig. 4, panel e1, while most D_x_(0.7) values were between 0.5 and 3 Gy in panel e7. This is also evident from the histograms in Fig. 3, however we see that the D_x_(0.1) values are unevenly distributed within this interval and the boundaries are not as clear as they seems to be in panel e1 of Fig. 4, while the trend becomes clearer for higher SFs with a distinct sharp fall in D_x_(0.7) values outside the interval 0.5–3 Gy. The RBE_0.1_ distribution (Fig. 3, panel d) has a clear peak between 1 and 1.1, with a sharp decrease, especially for lower values. For higher SFs, the most densely populated part of the distribution covers increasingly higher RBE values, and the data becomes more evenly distributed. While the peak is consistently found between 1 and 1.1, the mean RBE increases to 1.3 for SFs > 0.2 whereas the median RBE remains 1.2 across all SFs.

**Fig. 5:**
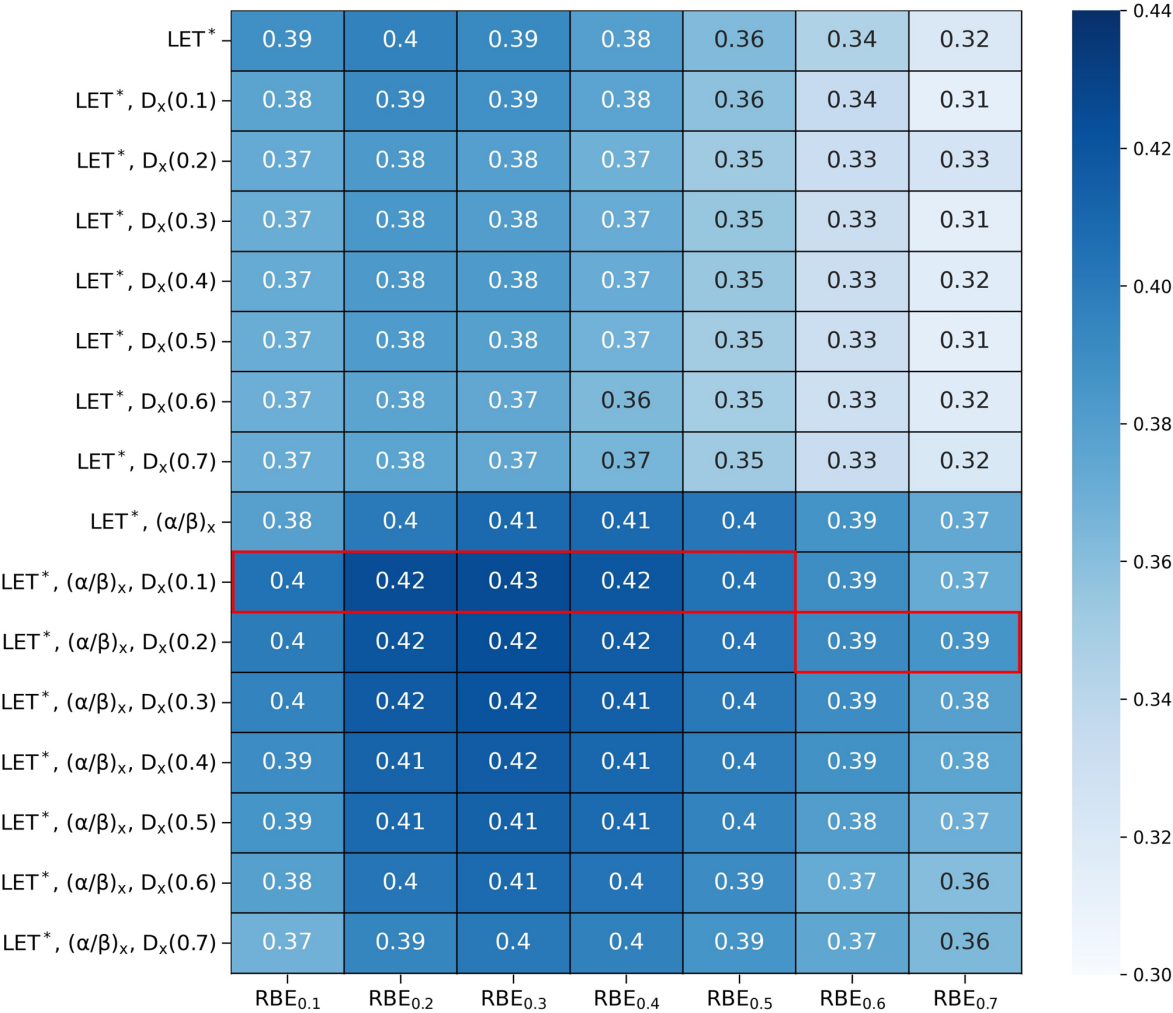
Heatmap showing R2 scores from the 4-fold cross validation on the training set for each considered inputs-output combination; RBE_SF_(LET), RBE_SF_[LET, D_x_(SF)], RBE_SF_(LET, (α/β)_x_) and RBE_SF_[LET, (α/β)_x_, D_x_(SF)], for SFs between 0.1–0.7. Inputs and outputs are given on the y- and x-axis, respectively. The highest R2 scores in each column, corresponding to best performing inputs for each output are highlighted by the red boxes.

Using D_x_(SF) as an additional input to LET and (α/β)_x_ gave increased cross validation R2 scores for all considered SFs (Fig. 5). Models of RBE_SF_[LET, (α/β)_x_, D_x_(SF)] also showed better performance than models of RBE_SF_[LET, D_x_(SF)], with increasing differences in R2 scores for RBE values at higher SFs. If we compare the RBE_SF_[LET, (α/β)_x_, D_x_(SF)] models to those with only LET as input, we see the same trend of higher scores for the prior which is more pronounced for RBE at higher SFs. Comparing inputs LET and D_x_(SF) versus LET and (α/β)_x_, the latter gave overall better mean R2 scores indicating that (α/β)_x_ is a better predictor variable compared to D_x_(SF), especially for high survival levels (low doses).

The best performing inputs for each RBE_SF_ used in the final seven models that were trained on the full training set and evaluated on the test set were LET, (α/β)_x_ and D_x_(0.1) for RBE_0.1−0.5_ and LET, (α/β)_x_ and D_x_(0.2) for RBE_0.6_ and RBE_0.7_ (red boxes in Fig. 5). The R2 scores of the models with all three parameters illustrate that these gave better predictions than the one- or two parameter models also after being penalized of the extra degree(s) of freedom. As seen in Fig. 5, for most RBE_SF_ values the highest cross validation score in this heatmap was obtained by more than one of the models with different inputs. In such cases of ambiguity, more decimals of the calculated R2 scores in Fig. 5 were considered to decide which of these inputs that would be used in the model evaluated on the test set. It should be noted that this is only a convenient way to choose between these models, as it is not expected that the chosen model will perform significantly better than the alternative models.

**Fig. 6:**
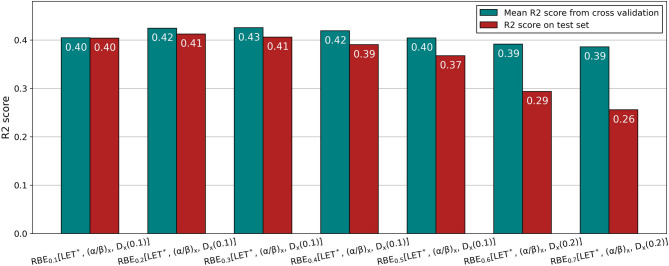
R2 scores from evaluation on the test set of models trained on the training data are given by the red bars. For each RBE_SF_, the inputs that showed best performance in the cross validation were used in the test-evaluation. For each model, the R2 score from the cross validation of the corresponding model (Fig. 5) is shown by a teal bar for comparison.

Evaluating the final models on the test set resulted in lower R2 scores compared to the mean scores from cross validation. The difference was minimal for the RBE_0.1_ model but increased for higher SFs with the RBE_0.7_ model yielding a 30% lower score on the test set (Fig. 6). Plotting predicted versus experimental RBE_SF_ values for the test data showed that the models performed well for low RBE_SF_s around and just above 1.0, while they struggled more with increasing RBE_SF_ where the predictions generally underestimated the experimentally derived values (Supplementary Figure A2).

**Fig. 7:**
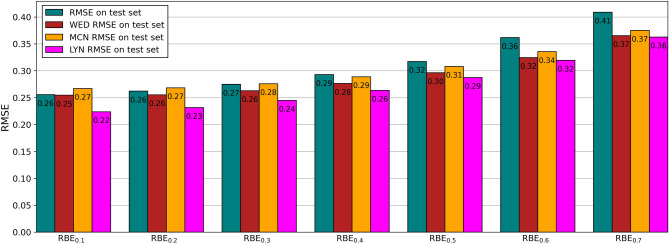
RMSEs of model predictions on the different RBE_SF_ test data. For each RBE_SF_, the teal bar represents the corresponding NN model from this work (as explicitly written on the x-axis in Fig. 6), while the red, orange and magenta bars represent the previously published WED, MCN and LYN models, respectively.

The NN models showed comparable performance as the published models on the test set for RBE_0.1−0.4_ and slightly worse for the RBE predictions at higher SFs (Fig. 7). As highlighted in the discussion, a comparison based on test RMSEs is likely biased in favor of the previously published models as parts of the test set were used to develop these, while for the NN models this is exclusively new data that was not seen during training.

**Fig. 8:**
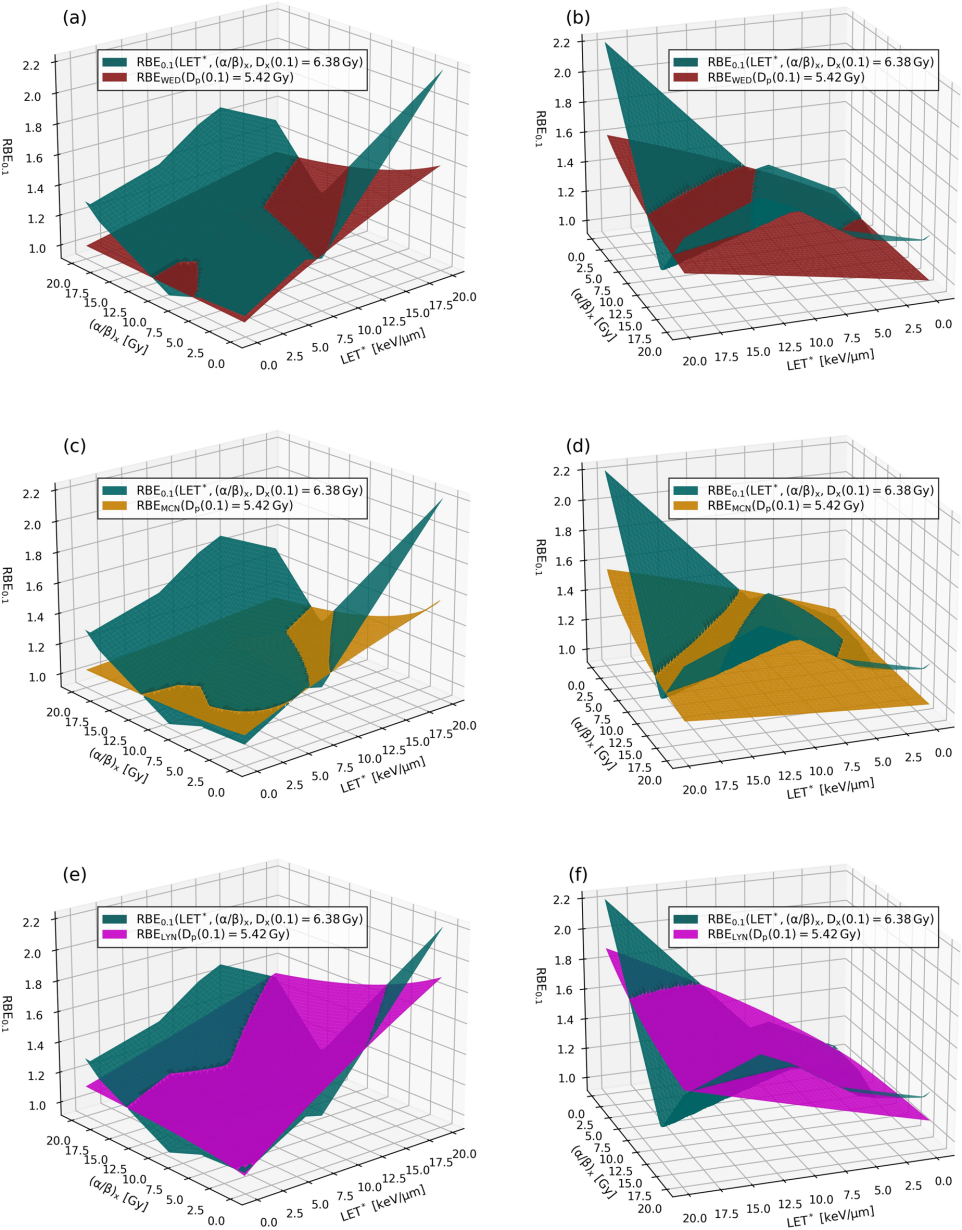
Planes showing predicted RBE_0.1_ as a function of LET and (α/β)_x_ according to the NN model from this work (teal) and the previously published WED, MCN and LYN models, shown in red (**a**, **b**), orange (**c**, **d**) and magenta (**e**, **f**), respectively. For each predicted plane, the dose input was set constant at the mean value of this parameter calculated for all data points in the database, given in the legend in each plot. The comparison is shown from two different angles (left and right panels).

Plotting predicted RBE_SF_ as a function of LET and (α/β)_x_ gave prediction planes of similar shape for all RBE_SF_ models. Predictions peaked at high LET combined with low (α/β)_x_ values, as seen for RBE_0.1_ in Fig. 8 and RBE_0.7_ in Supplementary Figure A3. The RBE_0.1_ model showed a sharp decrease in predicted RBE towards lower LET and higher (α/β)_x_, resulting in a valley of low predictions between LET = 20 keV/µm combined with (α/β)_x_ ≈ 14 Gy and LET around 7.5 keV/µm with (α/β)_x_ approaching zero (Fig. 8). This model also showed a distinct increase in predicted values from intermediate (α/β)_x_ towards higher (α/β)_x_ for all LET values. Similar trends were seen for all RBE_SF_ models, however, with increasing SF both the valley of low predictions and the increase towards high (α/β)_x_ became less pronounced, as can be seen for the RBE_0.7_ model shown in Supplementary Figure A3.

Compared to WED and MCN, the RBE_0.1_ NN model generally predicted higher RBE values (Fig. 8 panels a-d). However, compared to LYN this was only the case in the high LET/low (α/β)_x_ peak and for high (α/β)_x_, while for most of the considered parameter space the LYN model predictions were higher. Similar observations were made from comparing the NN models for RBE_0.2−0.5_ to the previously published models. However, observations differed in comparisons of prediction planes for RBE_0.6_ and RBE_0.7_, and especially notable was that LYN did not give higher RBE predictions than these NN models for most of the parameter space, as seen by comparing panels e and f in Supplementary Figure A3 and in Fig. 8.

## Discussion

We developed NN models for proton RBE values at specific SFs based on a comprehensive *in vitro* database collected from the literature. To minimize extrapolation beyond the experimental measurements, a suitable interval of SFs was found from the raw data provided in PIDE and applied to the database used in this study. The RBE_SF_ values were modelled according to a 4-fold cross validation scheme, using different combinations of input parameters to determine the best performing ones. Photon doses at specific SFs were included as inputs in the modelling, which combined with LET and (α/β)_x_ consistently yielded highest cross validation R2 scores across all modelled RBE values.

The raw data from PIDE demonstrated large variations in SF coverage between the *in vitro* experiments. For this study, the SF range from 0.1 to 0.7 was chosen as a compromise between being wide enough to model RBE at several distinctly different SFs while minimizing LQ curve extrapolation. Beyond this range there are fewer experimental data, increasing uncertainties in LQ parameter values and, consequently, in calculated doses and RBE_SF_ values.

Comparing the R2 scores in Fig. 5 obtained with LET and D_x_(SF) as inputs to corresponding scores for models that also takes (α/β)_x_ as input, revealed a trend of the latter group performing increasingly better for higher SFs. As a higher SF is equivalent to lower dose, this can be seen as moving towards RBE_max_ and away from RBE_min_, and these results thus indicate that (α/β)_x_ is most influential for the RBE_max_. This is in line with previous results from RBE_max_- and RBE_min_ based modelling, indicating that the RBE_max_ function has a strong dependency with (α/β)_x_, while the RBE_min_-(α/β)_x_ dependency is considerably weaker, or even insignificant^16^. Models with LET, (α/β)_x_ and D_x_(SF) as inputs also showed consistently better performance than corresponding models with only LET and (α/β)_x_ as inputs (Fig. 5). As the calculated R2 scores were adjusted for inclusion of additional input parameters in the model, this shows a clear trend that the D_x_(SF) input provided additional information in the modelling, and it is thus generally beneficial to include this as an extra parameter in the models. Moreover, studying the last seven rows in Fig. 5 further, there is a systematic trend of decreasing R2 scores going down each column, indicating that photon doses at lower SFs are consistently better input parameters than those at higher SFs. This is also reflected by the final inputs used to model each RBE_SF_ based on best cross validation performance, including D_x_(0.1) for modelling of RBE_SF_ at SFs 0.1–0.5 and D_x_(0.2) in the RBE_0.6_ and RBE_0.7_ models. The increased performance of photon dose inputs at lower SFs may be connected to the larger spread of parameter values compared to doses at higher SFs (Fig. 3, panel c vs. panel o), as the model is generally able to learn more from input parameters with broader distributions. These results indicate that to establish a standardized input parameter for the photon dose at a specific SF, an appropriate choice of SF could be in the range 0.1–0.2. Although the present analysis focused on exploring the D_x_(SF) input, exploration of α_x_ and β_x_ as modelling inputs could be an interesting objective for future work. While the concept of specific SFs is less useful in a clinical setting, taking the negative natural logarithm of SF and dividing it by α_x_ provides the biologically effective dose (BED), which has many applications. Further, the BED can be converted to obtain the equivalent dose in 2 Gy fractions (EQD2), a useful metric for clinical scenarios.

The amount of data decreases drastically with increasing LET and (α/β)_x_, meaning that each of these data points is more influential for the fitting of the model in these regions, compared to each data point in the more abundant region of the parameter space at lower LET and (α/β)_x_. Data with higher LET may also be more prone to errors as these experiments are harder to perform accurately, which would result in more variability in such data. The shape of the fitted model in these regions may therefore be largely determined by the randomly selected data used for training. When evaluating the model on unseen data, the R2 score may then depend heavily on the distribution of these data in relation to the training data. For example, if the unseen data used for evaluation contains a larger proportion of data with higher LET and (α/β)_x_, this may have a negative impact on the score. On the other hand, a lower proportion of such data can lead to a higher score, as less data are considered in regions where the predictions are associated with larger errors.

The cross-validation strategy is intended to counteract such effects of random variations when splitting the data by performing several splits and evaluating based on the mean score across all of them. However, the modelling and evaluation in each fold can be affected by the smaller portion of data with higher LET and (α/β)_x_ which likely differ more between splits compared to the much more abundant and consistent data at lower LET/(α/β)_x_. Stratification of these variables could be an option, introducing restrictions in the data splitting to ensure similar distributions in each subset. However, this will introduce bias in the modelling procedure and should be done with extreme care as randomness is a key feature of the ML workflow. An alternative strategy could be to create a more balanced dataset by randomly sampling a fixed number of points from different regions of the parameter space. However, dividing the space into regions that ensure balance across all parameters is challenging. Also, sparse regions limit sample sizes, leading to a smaller dataset less suited for ML modelling. Reusing data from sparse regions could enlarge the dataset but results in reused points being more heavily weighted in the modelling, introducing bias, potentially towards data with larger uncertainties.

A NN with two hidden layers of size 6 was used by Tian and Lühr^23^ to model *in vitro* RBE data from a range of different ion species, resulting in R2 scores on the test set between 0.74 and 0.77 for prediction of RBE at a reference photon dose of 2 Gy. This is notably higher than the R2 scores between 0.26 and 0.41 achieved with the present proton-specific models. However, Tian and Lühr considered test data from different ions, while no proton-specific test score was reported. The present NN models showed comparable or slightly lower performance on the test data compared to previously published models. ML models should be more flexible than regression models based on predefined fitting functions, as the relationships between different parameters are determined directly from the training data instead of obeying a set of assumptions for the dependencies that are made prior to model fitting. The LYN and MCN model databases contained 98% and 68% of the test data, respectively, meaning that a fair comparison to our model, for which the whole test set is new and unseen data, is not possible. On the other hand, only one data point in the test set was used to develop the WED model. However, while the MCN and LYN models are based on more than 250 and 450 data points, respectively, the WED model database only contains around 20 data points. An alternative approach to model comparison would be to refit the LYN, MCN, and WED models to the training data and use these refitted models for test predictions. However, to maintain relevance and comparability with the literature, the evaluation was based on the original published models. This approach is also consistent with recent studies proposing new RBE models, which commonly compare their results against the original versions of previously published models.

The NN model prediction planes in Fig. 8 and Supplementary Figure A3 are not smoothly curved planes but seem to be composed of relatively flat sub-planes with rather abrupt changes between them, forming a landscape with sharp edges and peaks. This could be caused by regions of few data, where the model simply does not have enough information to form a smooth curve between the points. However, it could also be a result of implementing a too simple model. In this work, NN models with a maximum depth of three layers were considered as candidates for model selection, while it could be that more layers are needed to model the data. On the other hand, overfitting might become a problem if the model becomes too complex, which will likely happen if the number of layers in the NN is significantly higher than the number of input parameters. In this regard, for modelling with one, two or three input parameters, it can be argued that NNs with depths between 1 and 3 layers should be sufficiently flexible while avoiding overfitting due to overly complex models. As was seen in Fig. 8 and Supplementary Figure A3, the differences between the ML models and the published models were largest for higher LET and (α/β)_x_. It might thus seem like the general trend in these regions with less data can be better captured by the assumption-based regression procedure applied in the previously published models compared to the current ML strategy. In this case, acquiring more data in these sparse regions will be a prerequisite for future assumption-free proton RBE modelling.

The fact that experiments have been conducted at different institutions is a major contributor to the observed data variability and imbalanced distributions, as no standardization of experimental procedures and error reporting have been established and followed across institutions. There are thus severe limitations to what information can be gained from a large multi-institutional dataset due to these inherent variations^41^. One potential solution to this could be to perform many cell irradiation experiments in a single laboratory, using the same setup and reference radiation for a wide range of cell lines. Analysis of such a large and diverse dataset obtained under consistent experimental conditions would be more statistically robust, and likely contribute to advancing the understanding of variable proton RBE and its influencing factors. However, restricting the analysis to data obtained at a single institution may introduce systematic errors that cannot be identified through analysis of these data alone but only through comparison with other datasets.

## Conclusion

Our study demonstrated that NN modelling of proton RBE, applied to data within a restricted range of SFs where experimental raw data are most abundant, offers an assumption-free approach to RBE modelling. Including the photon dose at specific SFs as a predictor variable improved the prediction power of the resulting RBE models, favoring doses at lower SFs witch showed distinctly broader distributions than higher SF-doses. The presented approach addressed extrapolation limitations inherent in traditional RBE_max_ and RBE_min_ regression-based models while demonstrating comparable RMSE performance. However, the reliability of assumption-free RBE modelling remains constrained by data sparsity in specific regions, emphasizing the need for further experimental data and large, diverse datasets acquired under consistent experimental conditions to fully realize its potential.

## Supplementary Information

Below is the link to the electronic supplementary material.

Supplementary Material 1

## Data Availability

The files of the newest PIDE version are available upon request at: [https://www.gsi.de/en/work/forschung/biophysik/forschungsfelder/radiobiological\_modelling/pide\_project](https:/www.gsi.de/en/work/forschung/biophysik/forschungsfelder/radiobiological_modelling/pide_project). The proton RBE *in vitro* database that was used in this study is available in the publication by Lyngholm et al. from 2024^16^.
