# Supplementary material for: Neural network modelling of proton RBE values at predominant survival fractions of in vitro data
Source: Sci Rep. 2026 May 22;16:23385. doi: 10.1038/s41598-026-54123-w (PMC13408797; doi:10.1038/s41598-026-54123-w)
Supplement: Supplementary file 1 — Supplementary Material 1 [file 41598_2026_54123_MOESM1_ESM.pdf]

Neural network modelling of proton RBE values at predominant survival fractions of in vitro data

Erlend Lyngholm*[a], Armin Lühr[b], Liheng Tian[b], Camilla Hanquist Stokkevåg[a, c], Helge Henjum[a], Andreas Havsgård Handeland[a, c], Johannes Tjelta[a, c], Kristian Smeland Ytre-Hauge[a]

a Department of Physics and Technology, University of Bergen, Bergen, Norway
b Department of Physics, TU Dortmund University, Dortmund, Germany
c Cancer Clinic, Haukeland University Hospital, Bergen, Norway

*Corresponding Author email address: erlend.lyngholm@uib.no

Supplementary information

LET_d normalization

The database contained proton RBE values relative to photons of different radiation qualities. The photon energies are subject to different LET values, and thus have an RBEx relative to each other, which must be considered when pooling these data. To account for this, the proton LETd values were normalized relative to the LETd of Cobalt-60 using [1,2]

$$\mathrm{LET}^* = \mathrm{LET_p} - \mathrm{LET_x} + \mathrm{LET}_{^{60}\mathrm{Co}}, \quad \text{(A1)}$$

where LET* is the normalized LET_d value, and LET_p, LET_x and $LET_{^{60}Co}$ are the LET_d values of the protons, reference photons and Cobalt-60, respectively. LET_x values were extracted from Howard (2017) [3] and Mairani et al. (2016) [2] (Table A1). Missing values for kV X-rays were estimated by linear inter-/extrapolation, and the LET_x for 6 MV photons adapted from Howard (2017) [3] was assumed valid for all MV-range photons (Table A1).

Table A1: LET_x values used to normalize proton LET_d values to the LET_d of ^{60}Co γ-rays. Additional LET_x values from Mairani et al. (2016) [2] that were used in the linear interpolation to obtain missing values are also given (100- and 220 kVp X-rays).

Reference radiation	LET_x [keV/µm]	Reference
^{60}Co γ-rays	0.4	Mairani et al. (2016) [2]
^{137}Cs γ-rays	0.8	Howard (2017) [3]
100 kVp X-rays	1.443	Mairani et al. (2016) [2]
120 kVp X-rays	1.387	From linear interpolation
130 kVp X-rays	1.359	From linear interpolation
180 kVp X-rays	1.220	From linear interpolation
200 kVp X-rays	1.164	Mairani et al. (2016) [2]
220 kVp X-rays	1.127	Mairani et al. (2016) [2]
225 kVp X-rays	1.118	From linear interpolation
240 kVp X-rays	1.092	Mairani et al. (2016) [2]
250 kVp X-rays	1.075	Mairani et al. (2016) [2]
300 kVp X-rays	0.990	From linear extrapolation
4-, 6-, and 10 MV photons	0.2	Howard (2017) [3]

Coefficient of determination

A data set with n values can be written as a vector **y** = [y_1, …, y_n]. If each value is associated with a predicted value in the vector **f** = [f_1, …, f_n], we can define the residuals as $e_i = y_i - f_i$, forming a vector **e**. The residual sum of squares and the total sum of squares are given as

$$SS_{res} = \sum_i (y_i - f_i)^2 \tag{A2}$$

and

$$SS_{tot} = \sum_i (y_i - \bar{y})^2 \tag{A3}$$

respectively, where $\bar{y}$ is the mean of the observed data. The most general definition of the coefficient of determination (R2 score) is

$$R^2 = 1 - \frac{SS_{res}}{SS_{tot}} \tag{A4}$$

To account for the phenomenon of the R2 score automatically increasing when extra input variables are included in the model, the adjusted R2 can be used, given as

$$\bar{R}^2 = 1 - \frac{SS_{res}/df_{res}}{SS_{tot}/df_{tot}} \tag{A5}$$

where df_{res} is the degrees of freedom of the estimate of the population variance around the model, and df_{tot} is the degrees of freedom of the estimate of the population variance around the mean. df_{res} is given in terms of the sample size, n, and the number of variables, p, in the model, $df_{res} = n - p - 1$, and df_{tot} is given in the same way, but with p being zero for the mean, $df_{tot} = n - 1$.

For prediction evaluation, e.g. a model trained on a training set and evaluated on a test set, the out-of-sample (OOS) R2 score should be used, where $\bar{y}$ in SS_{tot} represents the mean of the training data instead of the mean of the OOS test data when calculating the R2 score according to equation (A4).

Combining the above, the adjusted OOS R2 score used for prediction evaluation in the cross validation and for evaluation of trained models on the test set in the current study can be written as

$$\bar{R}^2_{OOS} = 1 - (1 - R^2_{OOS})\frac{n-1}{n-p-1} \tag{A6}$$

Supplementary tables

Table A2: For each hyperparameter considered in the 4-fold cross validation scheme on the training data, all the investigated parameter values are given.

Hyperparameter	Parameter values investigated in cross validation
Hidden_layer_sizes	(n), (n, m), (n, m, r), where n, m and r ∈ [1, 6]
Activation	‘tanh’, ‘relu’
Solver	‘sgd’, ‘adam’
Alpha	0.00001, 0.001, 1
Random_state	0, 50, 100, 150, 200, 250, 300, 350, 400, 450

Table A3: Hyperparameters and inputs used in the final models for each RBE_{SF} are listed along with the corresponding mean R2 scores from the cross validation.

Output	Best inputs	Best hyperparameters					Mean R2 score from cross validation
		Hidden_layer_sizes	Activation	Solver	Alpha	Random_state	
$RBE_{0.1}$	LET, $(\alpha/\beta)_x$, $D_{x,0.1}$	(3, 6, 3)	'relu'	'adam'	1.0	200	0.404
$RBE_{0.2}$	LET, $(\alpha/\beta)_x$, $D_{x,0.1}$	(3, 6, 3)	'relu'	'adam'	1.0	200	0.424
$RBE_{0.3}$	LET, $(\alpha/\beta)_x$, $D_{x,0.1}$	(3, 6, 3)	'relu'	'adam'	1.0	200	0.425
$RBE_{0.4}$	LET, $(\alpha/\beta)_x$, $D_{x,0.1}$	(3, 6, 3)	'relu'	'adam'	1.0	200	0.419
$RBE_{0.5}$	LET, $(\alpha/\beta)_x$, $D_{x,0.1}$	(3, 6, 3)	'relu'	'adam'	1.0	200	0.404
$RBE_{0.6}$	LET, $(\alpha/\beta)_x$, $D_{x,0.2}$	(5, 6, 3)	'relu'	'adam'	0.00001	400	0.391
$RBE_{0.7}$	LET, $(\alpha/\beta)_x$, $D_{x,0.2}$	(5, 6, 3)	'relu'	'adam'	0.00001	400	0.386

Supplementary figures

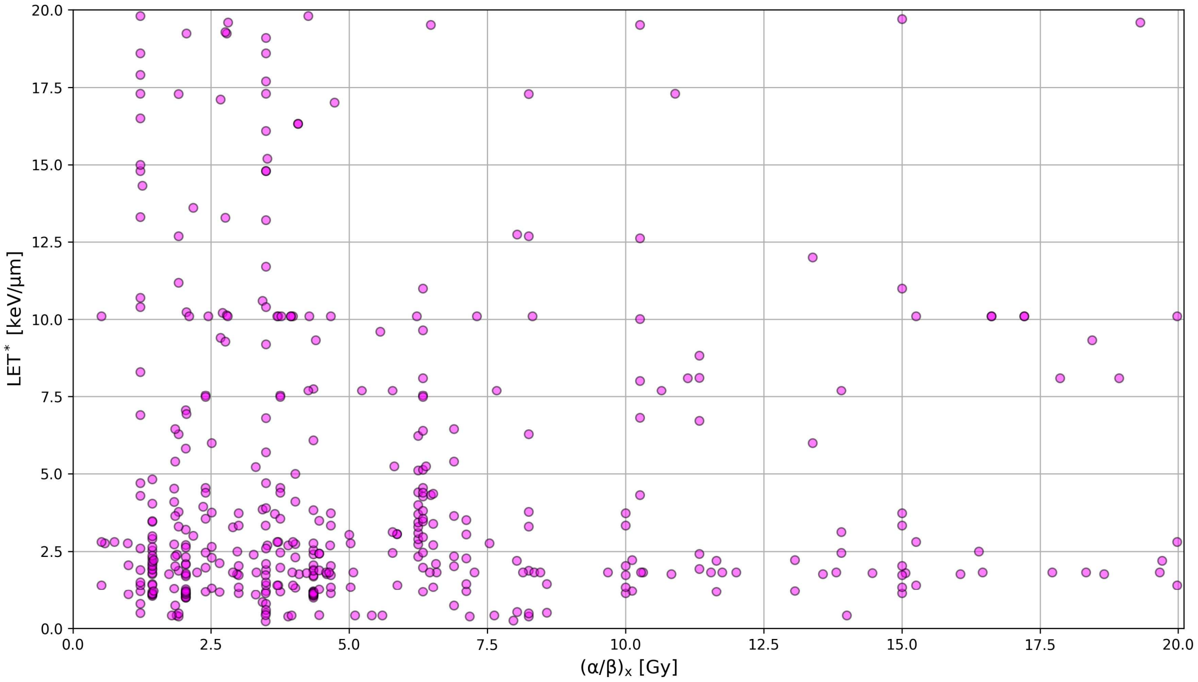

Figure A1: Scatterplot of LET vs $(\alpha/\beta)_x$ for all the 431 data points in the database.

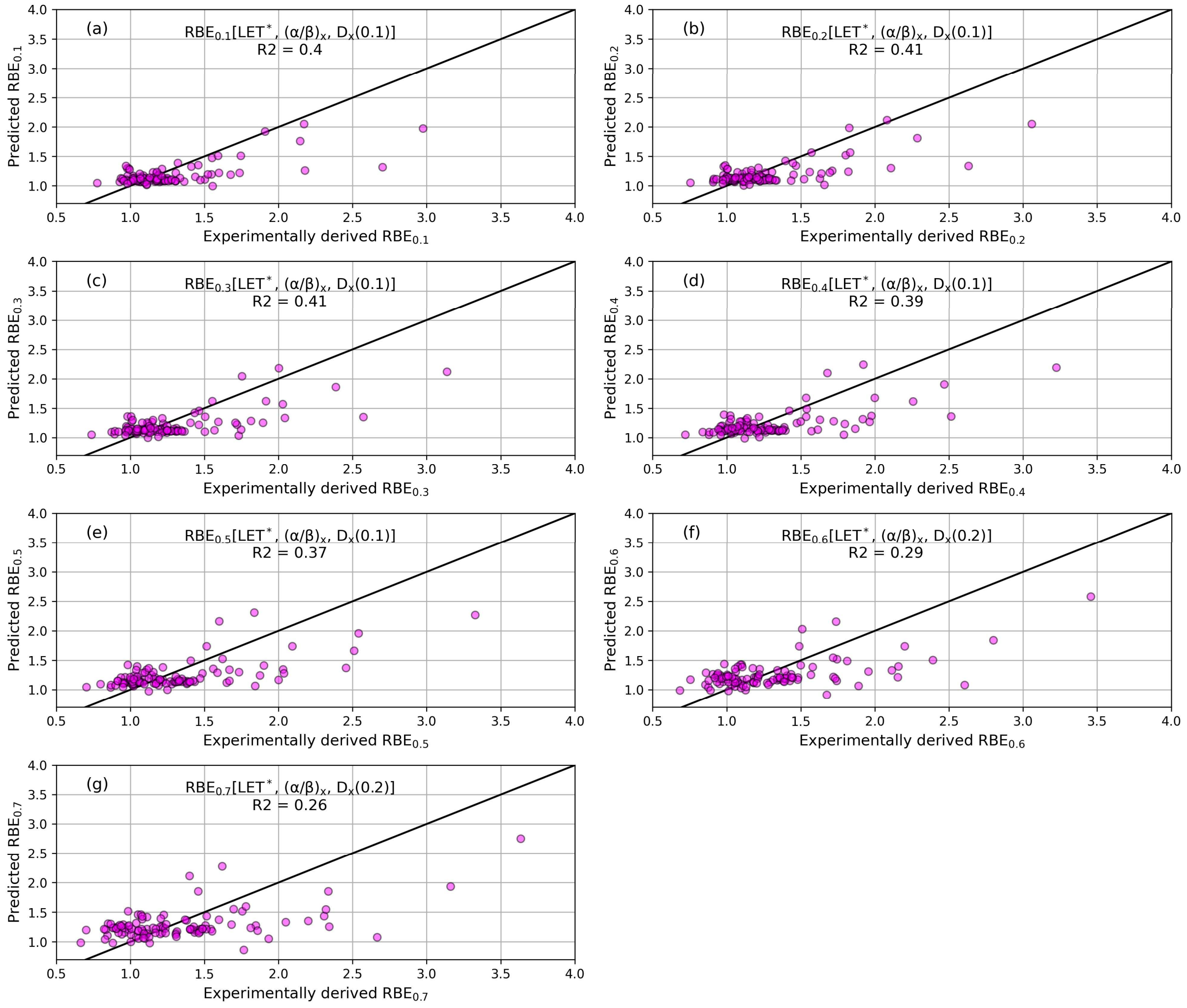

Figure A2: Comparisons between predicted and experimentally derived RBE_{SF} values for the test set are given for SFs 0.1 (a), 0.2 (b), 0.3 (c), 0.4 (d) 0.5 (e), 0.6 (f) and 0.7 (g). For reference, the black line in each plot shows the ideal case where predicted values are equal to the corresponding experimentally derived values (y=x). In each plot, the applied model that was trained on the full training set, as well as its R2 score from evaluation on the test data, are written at the top.

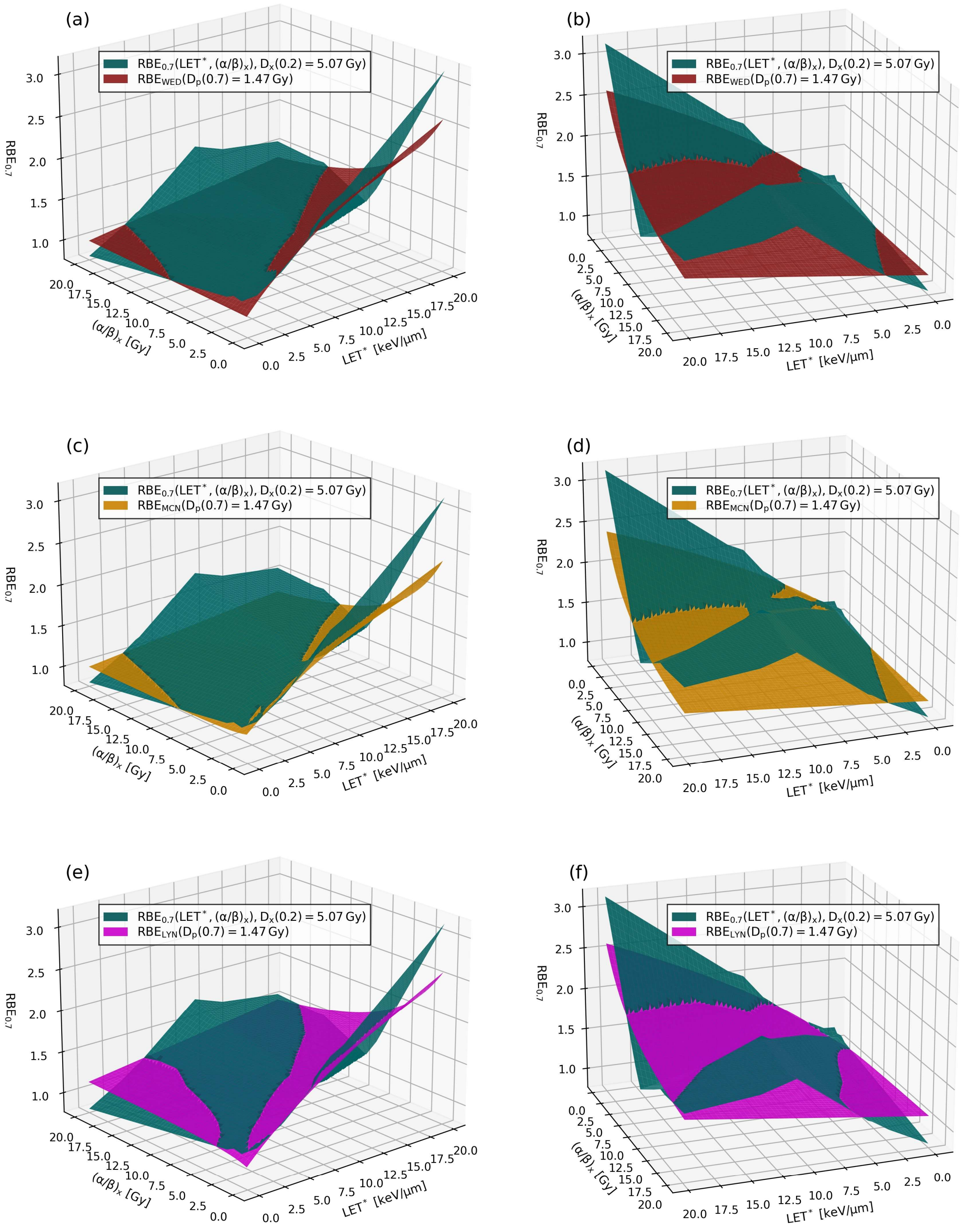

Figure A3: Planes showing predicted $RBE_{0.7}$ as a function of LET and $(\alpha/\beta)_x$ according to the NN model from this work (teal) and the previously published WED, MCN and LYN models, shown in red (a, b), orange (c, d) and magenta (e, f), respectively. For each predicted plane, the dose input was set constant at the mean value of this parameter calculated for all data points in the database, given in the legend in each plot. The comparison is shown from two different angles (left and right panels).

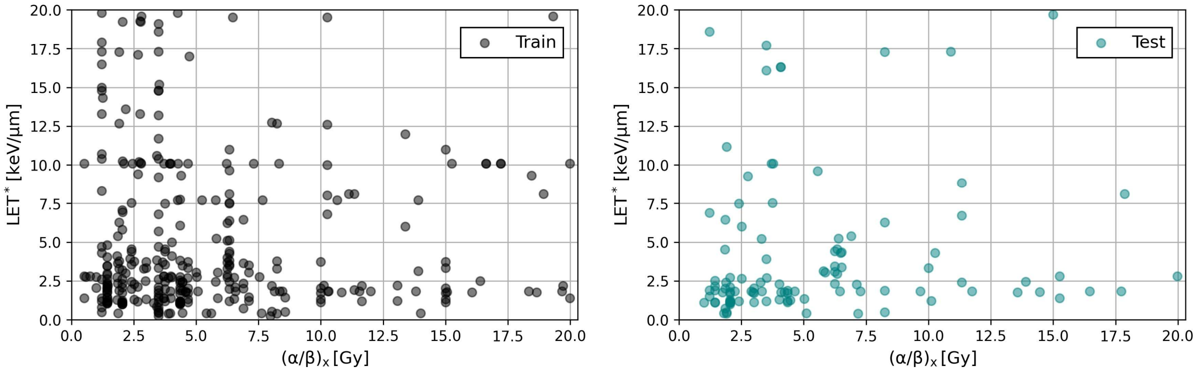

Figure A4: Scatterplots of LET vs $(\alpha/\beta)_x$ for the training set (left) and the test set (right).

References

1 Paganetti, H. Relative biological effectiveness (RBE) values for proton beam therapy. Variations as a function of biological endpoint, dose, and linear energy transfer. *Phys Med Biol* **59**, R419-R472, doi:https://doi.org/10.1088/0031-9155/59/22/r419 (2014).

2 Mairani, A. *et al.* Data-driven RBE parameterization for helium ion beams. *Phys Med Biol* **61**, 888-905, doi:https://doi.org/10.1088/0031-9155/61/2/888 (2016).

3 Howard, M. E. *Characterization of Relative Biological Effectiveness for Proton Therapy in Human Cancer Cell Lines* Ph.D thesis, College of Medicine - Mayo Clinic, (2017).
